# Recent Advances in the Non-viral Delivery of Genes to Central Nervous System Disorders

**DOI:** 10.1007/s10571-026-01703-z

**Published:** 2026-03-21

**Authors:** Hany E. Marei

**Affiliations:** Department of Cytology and Histology, Faculty of Veterinary Medicine, Mansoura University, Mansoura, 35116 Egypt

**Keywords:** Non-viral gene delivery, CNS disorders, Nanoparticles, Exosomes, Lipid-based delivery systems, BBB penetration, Gene therapy

## Abstract

Disorders of the central nervous system (CNS), neurological disorders, neurodegenerative disorders, genetic disorders) constitute a significant burden on global health, and current treatment options remain challenging. As treatment for CNS disorders is primarily palliative, the underlying causes of disease progression are not addressed through conventional pharmacologic therapies. Gene therapy has the potential to address these root causes of disease progression; however, many of the vectors used in gene therapy (e.g., adeno-associated viruses (AAVs)) have limitations such as immunogenicity, low cargo capacity, and crossing the blood-brain barrier (BBB). These limitations have led to significant progress in the development of non-viral gene delivery systems. Compared with viral vectors, non-viral platforms offer improved safety profiles, greater design flexibility, lower production costs, and superior suitability for repeated administration. This review reports recent advancements in the development of non-viral platforms for CNS gene delivery and focuses on lipid-based nanoparticles, polymeric nanoparticles, exosome-based techniques, and new hybrid technologies. Particular emphasis is placed on nanoparticle modification approaches to enhance BBB penetration and enable delivery of genome-editing technologies (CRISPR/Cas systems). The review provides explanations of clinical trials, regulatory considerations, and manufacturing issues that result from the recent developments noted above. It also explores the emerging role of artificial intelligence in supporting carrier design and enhancing delivery efficiency. Both artificial intelligence and non-viral platforms have the potential to facilitate the advancement of safe, effective, and repeatably administered gene therapies for patients with CNS disorders.

**Graphical Abstract:**

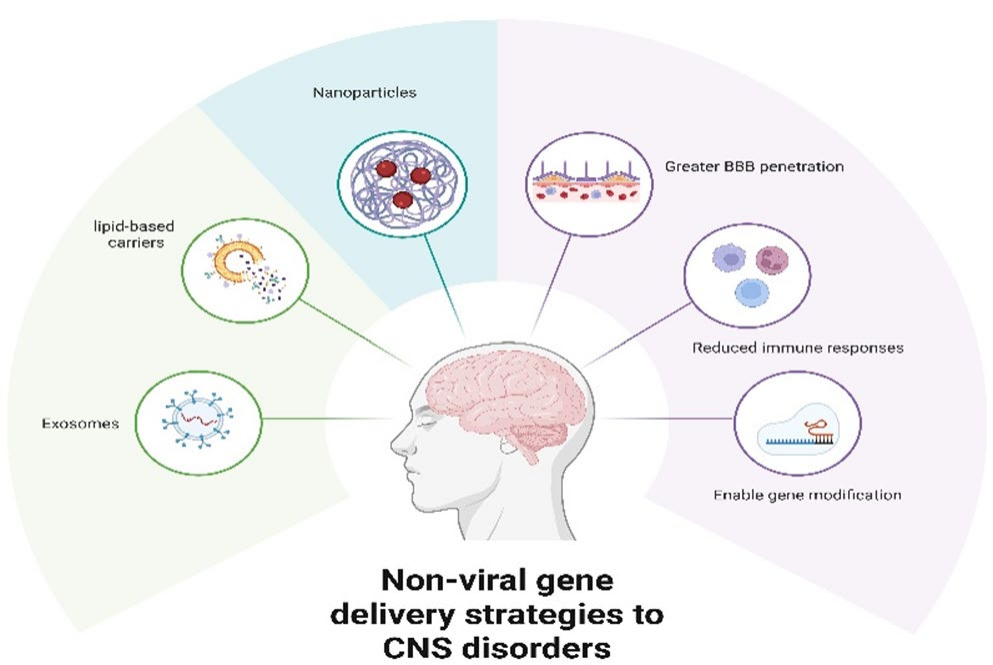

Nanoparticles, lipid-based carriers, and exosomes are examples of nonviral gene-delivery methods for CNS diseases. These systems are superior to viral vectors in several respects, including greater BBB penetration, reduced propensity to elicit immune responses, and the ability to enable gene modification (e.g., CRISPR-Cas9). This graphical summary illustrates the platforms, methods for crossing the BBB, and their advantages over the use of viral vectors.

## Introduction

The CNS, which includes the brain and spinal cord, is responsible for regulating many aspects of the body’s physiology. CNS-related health conditions, such as neurodegenerative disorders (i.e., Alzheimer’s Disease (AD), Parkinson’s Disease (PD)) and genetic health conditions (i.e., Huntington’s Disease (HD) and Spinal Muscular Atrophy (SMA), represent a significant burden on health systems around the world. Gene therapy is expected to provide new treatments that may have a longer-lasting effect by identifying and treating the underlying genetic causes of these diseases. Gene therapies currently use AAVs for gene transfer to the CNS. These viruses readily infect and express genes in the CNS. Still, they have limitations, including the risk of triggering an unwanted immune response, limited packaging capacity for the gene of interest, and difficulties transporting genes into the CNS across the BBB. Consequently, many researchers have begun developing non-viral gene-delivery vehicles to mitigate the limitations of AAVs while improving safety.

There are limited conventional pharmacologic therapies for CNS Disorders, including AD, PD, and HD, making the treatment of these disorders extremely difficult because the traditional pharmacologic therapies typically do not provide lasting benefits or prevent disease progression (Durães et al. 2018). Gene therapy is a feasible approach for delivering targeted treatments for neurological diseases via gene transfer technologies. The limitations of using viral vectors for gene delivery to treat CNS disorders will continue to encourage further research into non-infectious (alternative) systems to enhance their security, enable larger-scale production, and facilitate repeated patient administration (Dara et al. 2024).

The most successful methods for delivering genes into the CNS have evolved from viral vector-based approaches to non-viral methods, including nanoparticles (NPs), exosomes, and lipids. Modifying the physical and chemical characteristics of NPs to enhance cellular uptake and control the gene-delivery process is essential. The literature currently available indicates that lipid NPs (LNPs) made of bio-deoxidized lipids are capable of encapsulating and efficiently delivering ribonucleoproteins (RNPs) related to CRISPR-Cas9. The use of these NPs offers an alternative to other delivery methods and presents a unique opportunity for in situ genome modification with lower toxicity than traditional methods(Yang et al. 2024). Exosomes offer intriguing opportunities for researchers because they are biocompatible, naturally traverse the BBB, and can be engineered to allow targeted delivery of genetic material. The potential applications of exosome-mediated delivery of CRISPR-Cas9 for the treatment of neurological diseases represent a breakthrough in genetic engineering(Aslan et al. 2024). Magnetic nanocarrier systems are an emerging class of nanoparticle-based delivery platforms that may eventually prove useful for CNS-targeted gene therapy. In most cases, magnetic core materials, such as iron oxide-based nanoparticles, enable particle delivery using an externally applied magnetic field (Tomitaka et al. 2023). MNCs have been shown to improve targeting and cellular uptake, particularly when combined with a polymeric or lipid-based coating. Clinical translation of MNCs has been limited to date; however, increasing interest in using MNCs as externally controlled, image-guided nanocarrier platforms for CNS applications (e.g., studies employing magneto-electric nanocarriers) provides evidence of the emerging role of MNC technology as a viable platform for gene therapy delivery.

The information in Table 1 compares major non-viral gene delivery systems under development for Central Nervous System (CNS) applications, focusing on how the delivery systems work, how they are used to transport genes across the Blood-Brain Barrier (BBB), and what the advantages and disadvantages of each platform are based on the stage of development. As indicated by the comparison in Table 1, lipid-based nanoparticles and polymeric nanoparticles have the greatest potential for use as new treatment platforms for CNS gene therapy because they can be manufactured on a large scale, have controlled surface properties (surface charge or hydrophilicity), and have been shown to be safe for clinical use. In comparison, exosome-based delivery systems are relatively biocompatible and can generally penetrate the BBB, but they present challenges due to low production yields and heterogeneous product formation. Emerging gene delivery platforms (magnetic nanocarriers, hybrid nanoparticle systems) that can be externally controlled or guided by imaging have attracted increasing interest and prominence in the field; however, they remain primarily preclinical. Overall, Table 1 demonstrates that comparisons of delivery systems highlight the potential strengths and weaknesses of each platform and explain why no single non-viral delivery system has been shown to be universally applicable for CNS gene therapy at this time.

**Table 1:** Comparative Summary of Nanoparticles, Lipid Systems, Exosomes, and CRISPR-based Platforms for CNS Disorders

Dimension	Nanoparticles (NPs)(Saraiva et al. 2016b)	Lipid Systems (e.g., LNPs) (Hou	Exosomes(Alvarez-Erviti et al. 2011; Kalluri and LeBleu 2020a)	CRISPR-based Platforms (non-viral) (Duan
Definition	Engineered solid colloids (polymeric, inorganic, hybrid)	Lipid-based vesicular carriers, including solid lipid nanoparticles and lipid nanoparticles	Endogenous extracellular vesicles (~ 30–150 nm)	Platforms delivering CRISPR components (RNP, mRNA, gRNA) via non-viral carriers
Representative Materials	PLGA, PEI, chitosan, silica, gold	Ionizable lipids, PEGylated lipids, and solid lipid nanoparticles	Cell-derived membranes with intrinsic proteins	CRISPR–Cas9 RNPs, base editors, prime editors packaged in NPs, LNPs, or exosomes
Mechanistic Route Across BBB	Receptor-mediated transcytosis (RMT), adsorptive-mediated transcytosis (AMT), and size-dependent diffusion	RMT via ligand targeting, AMT; enhanced by PEGylation and surface targeting	Natural uptake pathways; likely RMT and membrane fusion; influenced by surface proteins	Dependent on carrier (LNP/NP/exosome); CRISPR cargo released intracellularly after BBB traversal
Cargo Types	Small molecules, nucleic acids (DNA, siRNA), proteins	RNA (mRNA, siRNA, gRNA), small molecules	Proteins, RNA, and lipids can deliver complex cargo	CRISPR RNPs, mRNA encoding editors, gRNA, donor templates
Cargo Capacity	Moderate to high (tunable)	High for RNAs; moderate for large proteins	Moderate; dictated by vesicle size and loading method	Variable; dependent on carrier and method (e.g., exosome vs. LNP)
Design Flexibility & Targeting	High; surface modification with ligands, peptides, aptamers	High ligand functionalization, PEG, targeted conjugates	High engineered surface proteins/ligands, hybrid vesicles	Very high; dual functionalization (carrier + CRISPR components)
Immunogenicity	Moderate; material-dependent	Low to moderate; minimized by PEG and biomimicry	Very low; inherently biocompatible	Low when delivered via exosomes/LNPs; minimal innate activation if optimized
Toxicity Concerns	Material-related (e.g., PEI cytotoxicity, inorganic cores)	Lipid composition influences toxicity; PEG hypersensitivity is possible	Very low; endogenous origin limits toxicity	Dependent on carrier (exosome best, NPs variable) and CRISPR off-target risks
Cellular Uptake	Efficient; enhanced by surface ligands	Efficient; dependent on lipid formulation and targeting	Highly efficient via natural uptake mechanisms	Highly efficient with engineered carriers; dependent on endosomal escape
Endosomal Escape Efficiency	Variable; often requires functional moieties	Generally high with ionizable lipids (e.g., ionizable LNPs)	Moderate; natural vesicle properties support cytosolic release	With optimized carriers (LNPs/exosomes) ≈ high critical for CRISPR activity
Scalability of Manufacturing	High (polymeric/inorganic standardization established)	Very high (LNP production for vaccines demonstrates scalability)	Challenging (isolation, yield, reproducibility)	Carrier-dependent (LNP scalable; exosome less so)
Regulatory Landscape & Clinical Stage	Early clinical (preclinical strong)	Advanced clinical (LNPs validated by mRNA vaccines)	Early clinical/preclinical; IND clearances emerging	Early translational; preclinical proof-of-concept for CNS applications
BBB Penetration Evidence	Preclinical models show penetration with targeting and small size (< 100 nm)	Strong evidence in preclinical CNS models; CNS delivery of RNA reported	BBB crossing in preclinical and nasally delivered studies	Demonstrated in preclinical studies with targeted carriers
Representative Applications in CNS	Nanoparticle DNA/siRNA delivery for neurodegeneration	LNP-mediated mRNA/siRNA delivery; CRISPR mRNA/RNP CNS editing	Exosome delivery of RNA/proteins; CRISPR cargo delivery	Exosome/LNP delivery of CRISPR to HD, AD, and PD models
Strengths	Highly tunable physiochemistry; robust manufacturing	Clinically validated backbone; excellent endosomal escape; high loading	Biocompatibility, inherent targeting, very low immunogenicity	Precision editing, versatile cargo, potential for disease modification
Limitations	Potential toxicity: BBB uptake varies with design	PEG immunogenicity; CNS targeting a work in progress	Manufacturing scalability, heterogeneity, cargo loading efficiency	Off-target editing; cargo stability; complex carrier optimization
Key Mechanistic Challenges	Balancing size/charge for BBB crossing vs. stability	Optimizing ligand targeting and minimizing immune clearance	Controlling cargo loading/release mechanisms precisely	Efficient editing with minimal off-target effects in CNS cells

Dendrimers and hydrogels/microspheres represent additional forms through which non-viral CNS delivery has been attempted. While dendrimers offer controlled architecture and surface modifications that enable efficient binding and cellular entry, their potential toxicity at high doses has raised significant concern. Hydrogels and microspheres have primarily been used for localized or sustained delivery rather than for enabling systemic crossing of the blood-brain barrier. Although the potential advantages of using liquid crystalline systems and noisome-based carriers for structural flexibility and enhanced drug stability suggest their likely use as methods for gene delivery to the CNS, their application for this purpose has not been widely demonstrated, as with lipid nanoparticles or exosomes, and therefore represents an area of development (Lin et al. 2024; Zhu et al. 2019).

**Fig. 1:**
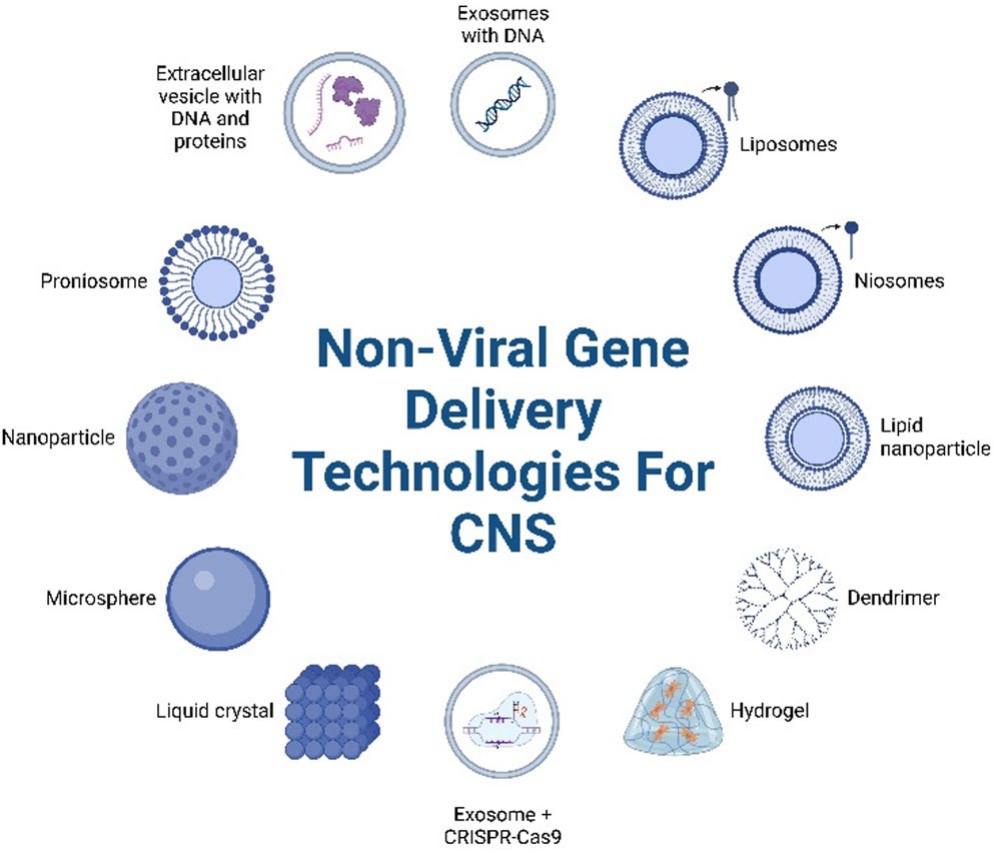
Non-Viral CNS Gene Delivery Systems. A schematic diagram of various non-viral gene delivery systems under investigation for use in the CNS is shown in Fig. 1. This diagram illustrates that there are a lot of different types of non-viral gene delivery systems, with many different types of devices that can carry DNA, mRNA, siRNA or CRISPR components. In addition, none of these non-viral gene delivery systems have as much immunogenicity, or can be modulated with surface functionality, or can be manufactured on a larger scale as compared with AAV-based gene delivery systems. Non-viral gene delivery systems that were not studied extensively prior to now are classified as “Emerging Systems,” whereas those that are widely studied as lipid or polymer carriers have substantial preclinical evidence before being used in humans

Delivery mechanisms are essential for gene therapy to reach target host cells. Gene therapy employs two methods for delivering therapeutic genes into host cells: viral and nonviral delivery systems. Viral delivery Mechanisms can be beneficial because they facilitate sustained and efficient expression of the therapeutic gene. Viral delivery systems (e.g., Adenovirus, lentivirus, Retrovirus) provide reliable transgene expression and a consistent response over time. Conversely, the advantages of using a non-viral delivery system include a lower potential to elicit an immune response (i.e., lower immunogenicity), a broader range of payload sizes (i.e., greater size flexibility), and a more favorable safety profile for human use. The application of exosomes in combination with CRISPR technology also opens up an entirely new approach for gene therapy by allowing scientists the ability to precisely alter the target gene, while at the same time reducing the likelihood of producing any unwanted side effects due to this alteration (i.e., lessening the risk of any “off-targets”) (Dara et al. 2024). Lipid nanocarriers can be used to create temporary gene expression. This eliminates the danger of inserting viral genetic material into a person’s own genome. These advances highlight how non-viral methods for delivering genetic material to the CNS could help address challenges currently facing CNS gene therapy and will ultimately be crucial for developing tailored treatments for neurodegenerative disorders. Non-viral gene delivery systems are becoming a viable alternative to viral systems. Examples include lipid nanoparticle (LNP) systems, polymeric systems, inorganic systems, exosomes, and hybrid systems. Each method has diverse structural features, loading capacity, targeting strategies and maturity of development for clinical use (Fig. 1).

Emphasizing their potential to address current challenges in gene therapy, this review provides a comprehensive, up-to-date overview of recent breakthroughs in non-viral gene delivery systems for CNS diseases. Unlike previous evaluations, this paper will incorporate new advances, including CRISPR-exosome hybrid systems, LNP-mediated precision genome editing, and creative approaches to enhance BBB penetration. Examining recent developments in clinical trials, regulatory challenges, and the expected integration of AI to optimize gene-delivery platforms will enable this review to critically evaluate the translational feasibility of non-viral systems. The goal of this review is to examine the creative aspects and to offer new perspectives, insights, and discussions, thereby providing a more thorough overview of the topic.

## Non-viral Gene delivery Methods

The rise of NPs as a method for the direct delivery of genetic material (i.e., DNA and/or RNA) to treat neurological disorders represents a promising opportunity for gene therapy. Because NPs can be tailored to their properties, they are rapidly becoming a key platform for the development of non-viral gene delivery systems. NPs can be modulated in size and surface properties, with important implications for their use as gene delivery systems(Ditto et al. 2009).

**Fig. 2:**
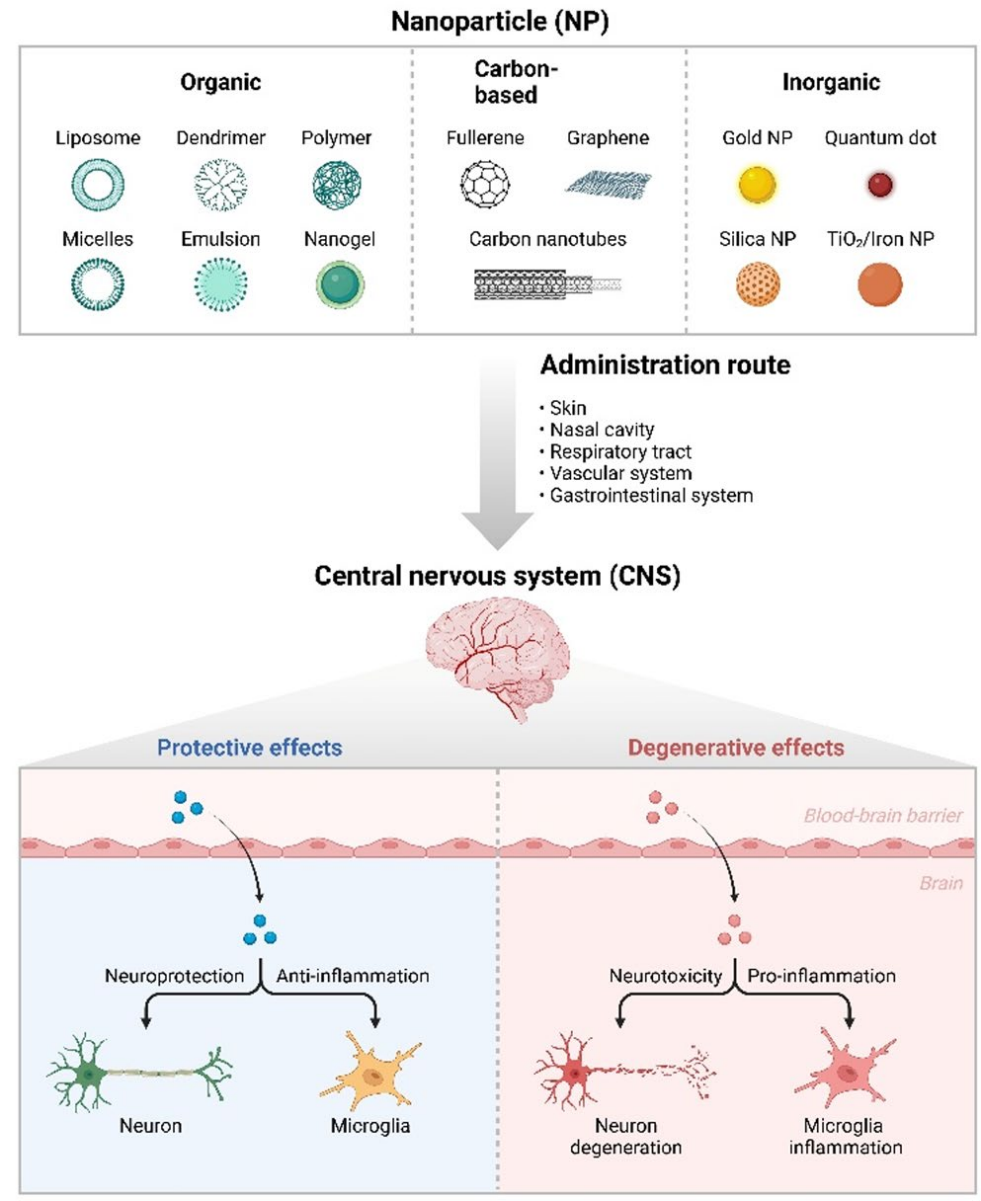
Variations in Nanoparticle Type Affecting the CNS. The major classes of nanoparticles are shown in Fig. 2 with the significant parameter for CNS targeting delivery due to biological barriers identified, including surface functionalization with targeting ligands (e.g., transferrin, ApoE peptides); PEGylation to prolong circulation time; optimization of size for enhanced transcytosis; and schematic representation of pathways for cellular uptake and intracellular trafficking of nucleic acids. These engineered characteristics are important for overcoming biological barriers and improving gene expression in neurons and glial cells. In particular, lipid-based delivery systems have demonstrated strong translational potential, owing to their use as mRNA platforms for vaccines and their modifiability to deliver RNA and CRISPRs. (Zhu et al. 2021)

NP-based approaches to delivering therapeutic genes into the CNS can be advantageous over other delivery methods because they can overcome barriers to achieving widespread CNS gene delivery. For example, some NP types not only encapsulate and protect the genetic payload from degradation or inactivation but also exhibita specific affinity for cells of interest. Through improvements, it appears likely that NP-based systems may help overcome significant challenges associated with treating CNS diseases with gene therapy, such as limiting the likelihood of an immune response being generated against transfected/gene-modified cells; enhancing the transfer/uptake of transferred genes into target cells; and improving the capacity for transferred genes to traverse the BBB(Mani et al. 2023) **(**Fig. 2**)**.

Several studies by different research groups have assessed the biodegradability and biocompatibility of polymeric NPs, including those based on Poly (Lactic-Co-Glycolic Acid) (PLGA), Polyethyleneimine (PEI), and Chitosan. To increase the cellular uptake and gene delivery efficiency of greater accuracy. Researchers can alter their physical properties (size, surface charge, etc.) and incorporate targeting ligands (transferrin, neurotrophic factors) to improve the accuracy of delivering therapies to the CNS (Dara et al. 2024; Mulvihill et al. 2020). Research indicates that PLGA NPs containing therapeutic genes are an effective means of delivering these genes to glial and neuronal cells, thereby enhancing disease model efficacy for both AD and PD. The use of PEI-based NPs for condensing genetic materials has been thoroughly investigated; however, the cytotoxicity of such NPs continues to be a challenge for the realization of clinical applications of PEI-based (Hauck and Hecker 2022).

LNP have been investigated for their unique compatibility with body tissues, their capacity to carry large amounts of genetic material, and their ability to cross the BBB; therefore, researchers consider them viable alternatives to viral vectors for gene transfer into the brain. Researchers have improved the delivery of small interfering RNA (siRNA) or DNA plasmids directly into the CNS by using liposomes (a type of LNP) modified with cationic lipids conjugated to brain-targeting antibodies or peptides. In addition, compared with liposomes, researchers are developing methods to precisely correct genetic disorders affecting the CNS using both solid LNP (another type of lipid-based NPs) and liposomes. This is due to the advantages of solid LNPs over liposomes, which offer greater stability and gene transfer efficiency (Aslan et al. 2024; Bashyal et al. 2022) Continued improvement of LNP formulations enhances their pharmacokinetic characteristics, thereby enabling a broader range of potential therapeutic applications.

Inorganic NPs exhibit good structural stability and ease of functionalization, and they can be coated to enhanced biocompatibility and BBB permeability. Delivery and imaging of genes using inorganic NPs occur less frequently than those using lipids and polymers; however, inorganic NPs offer an alternative for gene delivery and imaging (Cacciatore et al. 2016; Dara et al. 2024). Studies on gold NPs coupled with neurotrophic factors have shown that they can increase neuronal survival and enable regenerative mechanisms in models of neurodegenerative diseases (Khare et al. 2023).

Gene therapy may achieve greater and sustained efficacy through innovations that address issues related to cerebral circulation, e.g., methods and techniques that facilitate gene delivery across the BBB. In addition, the advantages and disadvantages of using vectors in gene therapy, and of both viral and non-viral vectors, have been addressed from a clinical perspective. They specifically discuss various approaches under development for exosome- and NP-mediated delivery of therapeutic genes, which may facilitate improved delivery of therapeutic proteins to the brain by reducing immunogenicity and increasing specificity for target cells. The authors also provide insight into additional issues affecting gene therapy in clinical practice for CNS conditions, including improved gene delivery efficacy, greater precision in targeting gene-delivery formulations, and compliance with regulatory protocols (Gao et al. 2024).

For intranasal delivery of plasmid DNA NPs, Harmon et al. (2014) evaluated their efficiency in transfecting and expressing reporter proteins in the rat brain, compared with other invasive delivery methods. According to the authors’ findings, this noninvasive delivery method increases the efficiency of gene delivery to the CNS. It enables more precise, localized transfection in distinct brain regions, producing reporter proteins. The authors highlight the potential of their work as a viable approach to targeted therapeutic intervention by introducing genes into the CNS via intranasal delivery systems for the treatment of neurodegenerative diseases and other CNS disorders (Harmon et al. 2014).

Marei (2022) discussed how multimodal targeting could be applied in the treatment of glioblastoma multiforme. Novel, specially designed nanocarriers capable of co-delivering multiple types of functionalized NPs using various biocompatible methods were presented. The co-delivery of NPs was intended to provide improved therapeutic efficacy and drug selectivity. The author also reported enhanced brain-cell targeting and fewer off-target effects by using various ligands, antibodies, and other biomolecules. Furthermore, he identified techniques that enabled enhanced targeting via magnetic or radiation-based delivery to the nanocarrier surface—moreover, functionalized NPs direct therapeutic agents, such as gene therapies or chemotherapeutic drugs, to the tumor site, thereby facilitating targeted delivery. This approach offers a promising way to improve CNS treatment, as it promotes greater drug accumulation in brain cells and helps sustain therapy (Marei 2022).

The most significant barrier to gene delivery to the brain via therapeutic approaches is the BBB. Several studies have assessed the ability of different types of NPs (liposomes, dendrimers, solid LNPs) to deliver their genetically loaded products to the CNS and have provided indications of an efficient manner for targeting the delivery of genetic information to the CNS. Advantages of utilizing NP-based delivery approaches include improved stability, reduced immunogenicity, and prolonged gene expression. Further research is needed to continue developing optimal NP design and delivery systems. As such, NP delivery systems have tremendous potential for use as an effective treatment option for patients suffering from neurodegenerative diseases and other disorders affecting the CNS (Annu et al. 2023).

In addition to virus-based therapies for CNS disorders, another non-viral treatment option is exosomes. Exosomes are an emerging area of research in CNS disorders and offer several advantages over traditional NP systems, including exceptional biocompatibility, efficient permeation across the BBB, and highly selective delivery of therapeutic or genetically engineered agents. Exosomes have several advantages compared with other NP delivery systems, but they also possess distinct physical properties that distinguish them from NPs and confer additional benefits. Exosomes provide a unique mechanism for intercellular signaling by transporting bioactive molecules and may reduce immunogenicity. These are some of the ways exosomes can be employed to deliver therapeutic drugs to the brain, and they also pose many obstacles that must be overcome before their full potential is realized in gene therapy approaches to CNS disorders (Avgoulas et al. 2023; Sharma and Mukhopadhyay 2024).

### Lipid-Based Delivery Systems

Lipid-based delivery technologies, particularly LNPs, have achieved significant clinical success owing to their use in mRNA vaccines. However, these lipid-based delivery technologies have transformed the field of non-viral gene therapy and also have numerous advantages, such as increased encapsulation capacity for nucleic acid, tunable physicochemical characteristics, and the ability to shield the DNA or RNA from degradation by enzymes in the bloodstream. The aforementioned characteristics of LNPs have made them the standard delivery vehicles for both RNA- and DNA-based therapies. In addition, for the treatment of CNS diseases, LNPs have been developed to maximize efficacy and enhance penetration across the BBB. Although macromolecules cannot be transported across the BBB, is promising (Ahmad and Pathak 2023). The versatility of magnetic nanocarrier (MNC) surface modifications enables targeting of specific cell types, thereby enhancing therapeutic efficacy in neurological diseases. Nanoparticles are now a key factor driving progress toward non-viral CNS gene therapies, owing to their tunable physical and chemical properties and their ability to protect nucleic acids from degradation. Nanoparticles made of a lipid bilayer, various polymers (such as PLGA, PEI, and chitosan), or inorganic substances can be designed for optimal cellular entry by varying particle size, surface charge (vl), and surface ligands, etc. (Fig. 2).

Recent years have seen a dramatic increase in interest in LNP technology for the delivery of therapeutic RNA agents (such as mRNA, siRNA, antisense oligonucleotides (AONs) into the human brain and other areas of the CNS. The most significant recent advancement in LNP technology was the development of synthetic ionizable lipids, which are neutral at physiological pH but positively charged under acidic conditions, such as those in the endosomal compartment after cells take up and process them. Ionizable lipids are thought to maximize the capacity of LNPs to promote gene expression after the release of mRNA into the cytoplasm following the endosomal escape processes. The remarkable success of COVID-19 vaccines formulated with LNPs and ionizable lipid-based mRNA has prompted renewed interest in developing therapeutic approaches to treat neurological diseases using similar formulations(Ahmad and Pathak 2023). Preclinical studies show that RNA molecules are readily delivered to the brain using LNPs formulated with lipids via ionization (both positively and negatively charged). The use of LNPs to deliver RNA produces high levels of gene expression in both glial and neuronal cells. across a wide range of CNS disorders (Buschmann et al. 2021).

Recent advances in RNA technology and lipid-based delivery vehicles have enabled many researchers to use these methods to introduce CRISPR-Cas9 and other gene-editing tools into the brain. Modifying or eliminating defective genes found in neuron cells will open a new frontier for treatment options for several neurodegenerative disorders, such as AD, PD, and ALS. Recent work has also shown that it is possible to use LNPs loaded with ribonucleoprotein (RNP) molecules containing the Cas9 enzyme to traverse the BBB and enable safe and effective gene editing “in vivo” within brain tissue. As a result of these findings, LNPs are attracting interest as potential delivery vehicles for CRISPR-based therapies (Farsani et al. 2024). Genetic factors may contribute to poor health outcomes over time and affect the expression of disease-related genes; however, both improved specificity and greater efficacy remain areas of ongoing research for CRISPR-LNPs. Continued development and refinement of lipid formulations and methods will be necessary to achieve targeted delivery of LNPs to appropriate cells (Sinclair et al. 2023).

Adding surface ligands to LNP formulations may enhance their ability to deliver to target sites in the CNS and facilitate cellular uptake of LNPs within the CNS. The addition of ligand molecules to low-density lipoprotein (LDL) formulations enhances LDL’s ability to cross the BBB and access the brain by acting as helper molecules. On the other hand, LDP pharmacokinetics can be modified and improved by polyethylene glycol (PEG) to prolong the circulation half-life and reduce clearance by the body’s immune system (Saraiva et al. 2016a; Zhang and Pardridge 2005). In the coming years, as scientists improve LNP formulation and delivery methods, lipid-based systems have the potential to replace traditional methods for treating CNS Diseases with gene therapy. Sophisticated lipid chemistry, combined with targeted ligands and gene-editing methods, will enable the development of alternative gene-therapy approaches that are effective and precise for treating neurological disorders.

### Exosomes in Non-viral Gene Delivery for CNS Disorders

In particular for CNS disorders, exosomes—tiny extracellular vesicles ranging in size from 30 to 150 nm—have emerged as a promising vehicle for non-viral gene delivery. Various kinds of cells generate vesicles for communication themselves, namely the transfer of substances such as proteins, lipids, or RNA molecules between cells. Exosomes can cross the BBB, a significant hurdle for many therapeutic approaches targeting CNS diseases and are therefore highly advantageous. Exosomes are biocompatible, have low immunogenicity, and can naturally interact with their target cells. Thus, exosomes are attractive candidates for gene therapy to treat neurological disorders, including glioblastomas, PD, and AD. The scientific and academic communities have shown increasing interest in exosomes as novel nonviral gene therapy delivery vectors for CNS disorders (Fayazi et al. 2021; Jarmalavičiūtė and Pivoriūnas 2016).

Exosomes may serve as a potential platform for therapeutic or gene therapy (e.g., recombinant DNA and RNA) and for gene-editing techniques (e.g., CRISPR), as they naturally form during evolution and adopt representative shapes. A lipid bilayer surrounds an exosome, creating a protective space for the genetic material to reach its destination. Moreover, engineered exosomes can be modified to incorporate molecular markers (e.g., ligands or antibodies) to direct them to receptors on cells infected with a virus (e.g., HIV) or that have begun to develop malignancies (i.e., cancer). By providing a targeted delivery system, this approach reduces the likelihood of adverse effects while increasing the therapeutic efficacy of the delivered genetic information. Exosome release from neural stem cells (NSCs) underscores the potential of exosomes as a therapeutic approach in preclinical trials of neurodegenerative models (Singh et al. 2024). Research has led to increased interest in using exosomes to treat CNS diseases non-virally as part of gene therapy (Pan et al. 2023).

Exosome-based gene delivery systems show considerable potential for clinical applications, but several challenges remain before they can be used as therapeutics. Current technologies for producing exosomes have low yield and scalability, which limit their use as therapeutics. Although exosomes are thought to cross the BBB, they are relatively ineffective at reaching their target sites. As such, further modification will be required for exosomes to fulfill their intended function within the CNS. Many strategies are currently being evaluated to enhance the production and transport of exosomes, including co-delivery systems using NPs that enable targeted delivery and improved absorption, as well as modifications to exosome permeability at the BBB. Before exosome-based gene therapies can be used in clinical practice, it is imperative to understand their form and function, and to ensure complete safety, including assessing potential risks and effects on the recipient, the immune response to the treatment, and any long-term effects (Batrakova and Kim 2015; Chen et al. 2024; Pishavar et al. 2022). Therefore, while exosomes show great promise as therapeutics for CNS disorders, these challenges must first be addressed before they can realize their full potential.

There has been a surge in recent years in studies investigating the development of improved methods to incorporate multiple types of molecules into exosomes for administration in biological systems. The primary focus of most research in this field has been on increasing the number of genetic products that an exosome can carry (i.e., the quantity of DNA, RNA, or other genetic materials) and on the extent to which the exosome can be integrated into the recipient organism. Additionally, methods have been developed to improve the stability and transportability of exosome-based products during storage and transit. The use of combinations of electroporation, sonication, and chemical modifications provides greater control over the customization of exosome physical properties, thereby improving their capacity to efficiently deliver genetic material to/from target cells. Additionally, manipulation of exosome surface components enhances their cell- or tissue-specific binding; thus, it provides greater precision in targeting the delivery of genetic material into the cell. Exosomes engineered with these technologies will enhance their function and provide greater therapeutic benefit for CNS Gene Therapy, creating a more useful clinical tool (Armstrong and Stevens 2018; Luan et al. 2017; Sun et al. 2022; Théry et al. 2002).

Combining therapies and approaches to maximize exosome delivery in CNS disorders is needed to maximize therapeutic benefit from exosome-based gene therapies. With advances in exosome-based delivery systems, RNA delivery methods (e.g., small interfering RNA), and genome-editing Tools (e.g., CRISPR/Cas9), these approaches will significantly enhance the efficacy of therapies for complex CNS disorders. Exosome-mediated transport of nucleic acids (DNA) and small molecules (including proteins) will enable a comprehensive therapeutic approach that targets multiple aspects of disease pathology simultaneously. Custom-engineering exosomes with various functional components opens new avenues for developing therapeutic strategies to treat individuals with CNS disorders. Numerous studies are currently evaluating new treatment methods, including exosome-based gene therapy for CNS disorders. Exosomes offer promise to both scientists and clinicians, as they enable the development of new treatment approaches for CNS Disorders(Kalluri and LeBleu 2020a; Li et al. 2024; Nouri et al. 2024; Perets et al. 2018; Shi et al. 2025a).

**Fig. 3:**
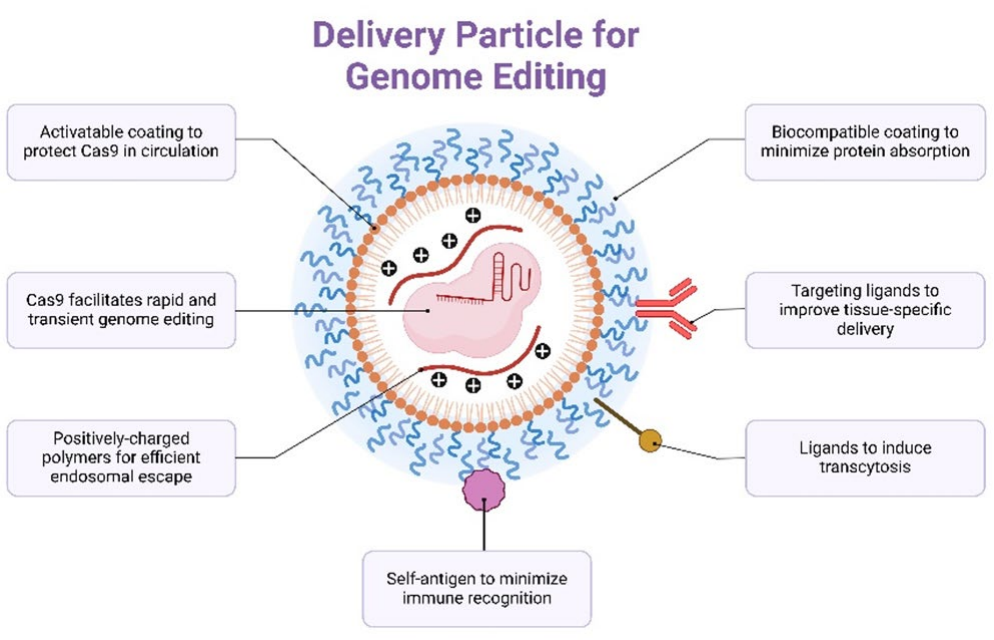
Genome Editing Delivery Systems. Design Features for a Nanoparticle-Based Delivery System with CRISPR-Cas9 Genome Editing Technology. This article depicts a multi-functional, non-viral delivery system designed specifically for CRISPR-Cas9 genome editing. An illustration is presented that highlights key elements of the design model, such as the incorporation of an activatable protective layer to prevent degradation of Cas9 during circulation; the use of positively charged polymer materials that can facilitate nucleic acid complexation and promote rapid and efficient endosomal escape; the addition of a biocompatible outer layer to decrease the likelihood of nonspecific protein binding; techniques used to target specific areas of the body leading to improved delivery efficiency and enhanced transcytosis of the nanoparticles through biological barriers, characterizing the additions of antigenic motifs to reduce the immune response and establish long-lived systemic stability for the delivery nanoparticles. (Tong et al. 2019)

Exosomes also provide a promising non-viral method for delivering genes to the CNS. While advances in exosome engineering may further rationalize this approach toward a viable therapy as an alternative to current techniques, the development of exosomes as a therapeutic option remains problematic in many cases for the reasons outlined above. Sustained research into and development of exosomes may provide new insights into their potential applications in CNS disease treatment and enable improved treatment strategies, benefiting a greater number of patients(Chen et al. 2024; Nouri et al. 2024; Shi et al. 2025a) (Fig. 3).

### CRISPR-Exosome Hybrid Systems

**Fig. 4:**
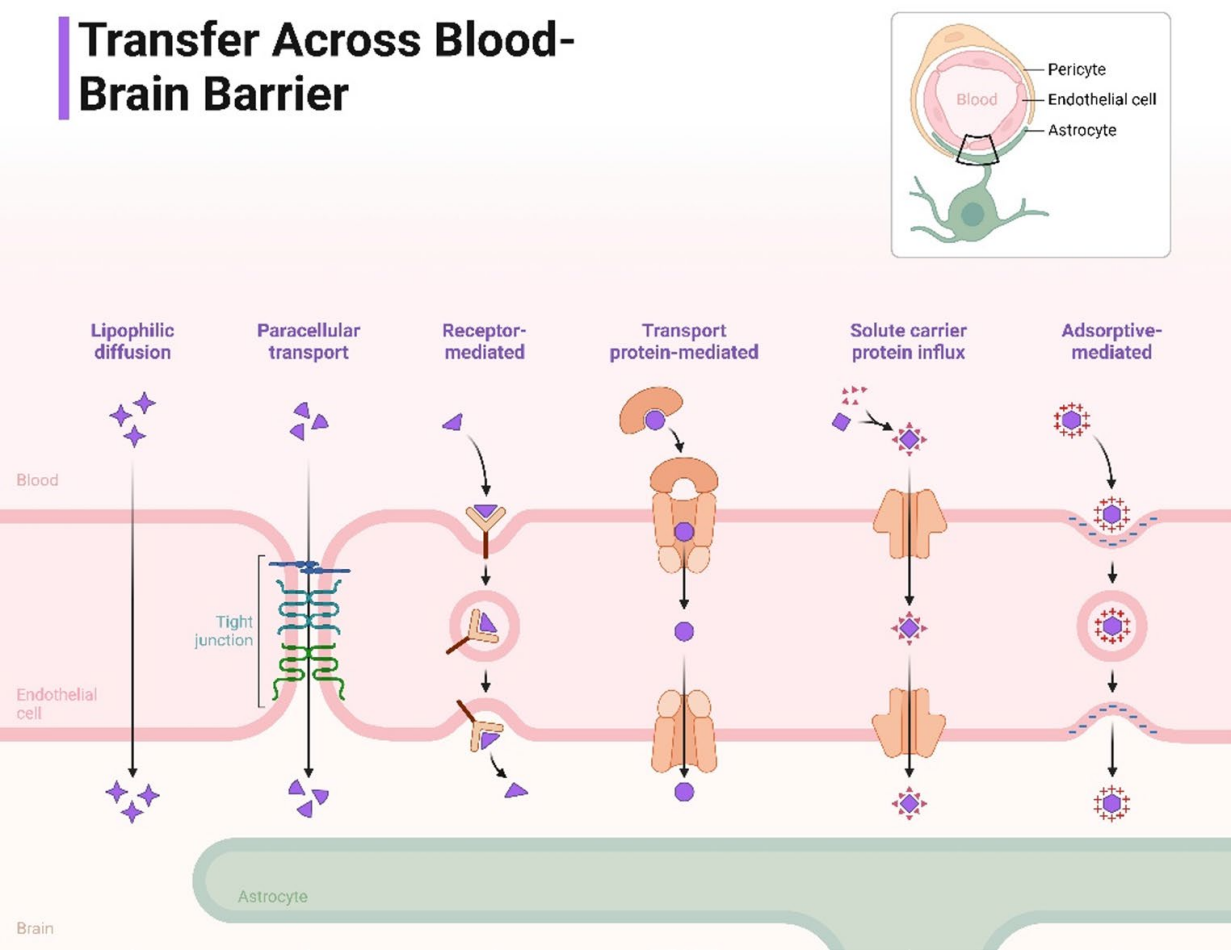
Translocation across the blood-brain barrier. Figure 3 shows the main ways in which engineered gene carriers cross the BBB. These are receptor-mediated transcytosis (RMT) or using receptors like transferrin or apolipoprotein E (ApoE); carrier-mediated transport with ligand conjugated nanoparticles; and adsorptive-mediated transcytosis, where the surface charge of the nanoparticle can affect how easily it will cross. There are also peptide-mediated transport methods, such as those using TAT or rabies virus glycoprotein (RVG) peptides, developed to promote cell uptake and BBB crossing. Physical methods, such as focused ultrasound (FUS) inducing temporary BBB disruption will increase the amount delivered by increasing the permeability of the BBB. The figure summarizes transport strategies telling how surface ligand modifications, optimize size of nanoparticles (especially less than 100 nm), and external physical modulation together maximize penetration of CNS (central nervous system). To provide for the rational design of gene carriers that optimize balance of efficiency and safety will require a good understanding of all pathways available. (Brookes et al. 2022; Nguyen et al. 2021)

The structure of neuronal networks and the BBB limit drug access to the CNS. Thus, CNS disorders, including neurodegeneration, e.g., AD, PD, and HD, become extremely challenging therapeutic opportunities. While CRISPR-Cas9 genome editing shows promise for gene therapy, its clinical application may be limited by off-target effects and delivery challenges(Doudna and Charpentier 2014; Salomonsson and Clelland 2024; Xie et al. 2023). Natural vehicles for CRISPR components have been examined, including exosomes—small extracellular vesicles with inherent biocompatibility and BBB permeability—which have yielded CRISPR-exosome hybrid systems(Duan et al. 2021a; Fatima et al. 2024; Yao et al. 2021). The use of CRISPR-exosome hybrids to treat CNS diseases offers several advantages, including their design features, systemic delivery methods, and the range of drugs that may be administered with these products. There are four of these transport mechanisms: (1) passive diffusion; (2) carrier-mediated transport, for example, through the use of receptors (including the use of exosomes as carrier vehicles); (3) receptor-mediated endocytosis (via receptors that specifically recognize the NPs); and (4) exosome-mediated transport. A fuller understanding of the potential of these devices as drug-delivery systems will require a sense of how these capabilities are enabled by the development of efficient drug-delivery devices for CNS diseases. Genome editing technologies such as CRISPR-Cas9 have the potential to change the way we can treat neurodegenerative and monogenic CNS disorders. A key challenge in using this technology remains the safe and effective delivery of CRISPR components to target cells. The use of exosome carriers has been identified as natural, biological carriers that can encapsulate both the Cas9 protein, guide RNA and donor templates. Exosomes also possess desirable characteristics such as biocompatibility and low immunogenicity. CRISPR-exosome hybrid systems enable precise gene editing through the enhanced natural transport capabilities of extracellular vehicles (EVs). This combination of technologies may reduce the risk of off-target effects, insertional mutagenesis, and transient modification of the target cell’s genome. (Fig. 4).

Exosomes are nano-sized vesicles produced by the exocytosis of cellular materials via multivesicular bodies. As such, exosomes can transfer biogenic molecules, including lipids and proteins, via intercellular networks to facilitate intercellular communication (Zeng et al. 2022). Their ability to pass the BBB makes them attractive targets for CRISPR-Cas9 delivery (Alvarez-Erviti et al. 2011). The CRISPR/exosomes delivery system uses exosome encapsulation combined with a CRISPR system that contains Cas9 protein, a gRNA, and donor DNA templates for HDR (homology-directed repair) to facilitate the cell’s uptake of CRISPR materials through electroporation, cell transfection, and other methods that will effectively load the exosome with CRISPR components (Bashyal et al. 2022).

Off-target effects of CRISPR/Cas9 have proven to be one of the most significant challenges in CRISPR-based therapies under development. The authors propose that exosomes should be used as delivery vehicles to target cells and to administer exosome-derived drugs directly, thereby significantly reducing the likelihood of Off-Target effects (Berggreen et al. 2023; Haizan et al. 2023; Zhu et al. 2023). Oligonucleotides can be incorporated into engineered exosomes, enabling ligand-target display on their surfaces to enhance cell communication (Akyuz et al. 2024a). By leveraging an engineered production platform, exosomes may address challenges associated with viral vectors, thereby reducing the likelihood of immune reactions. Furthermore, because they are not associated with viral vectors, exosomes do not pose a risk of insertional mutation (Mendell et al. 2017), by providing a non-immunogenic medium for CRISPR distribution. Their small size (30–150 nm) and natural origin help prevent phagocytic clearance, thereby increasing their circulation lifetime in the bloodstream. (György et al. 2014). Exosome-mediated delivery of CRISPR-Cas9 components has been shown to significantly alter gene expression in the brain (Dubey et al. 2024; O’Brien et al. 2020; Shi et al. 2025b).

Engineered exosomes that include CRISPR-Cas9 to eliminate the APP (amyloid precursor protein) stored in the brains of mice with AD significantly decrease the amount of amyloid found in these brains. This finding supports the idea that engineered exosome technology offers new possible avenues for treating this debilitating disorder (Akyuz et al. 2024a; Han et al. 2024). Exosome-mediated CRISPR Gene Editing for SNCA (α-synuclein) has the potential to protect against neurotoxicity in PD. The authors believe that, given the encouraging results from these studies, exosomic gene editing should be considered for the treatment of CNS diseases (Aslan et al. 2024; Kong et al. 2024; Mehdizadeh et al. 2025a).

Researchers have long been interested in genes implicated in the development of amyloid-beta (Aβ) and tau-mediated disorders. The potential use of CRISPR delivery via exosomes as an innovative therapeutic approach for AD is a relatively new idea. Recent preclinical studies have shown that exosome-mediated delivery of CRISPR/Cas9 targeting the BACE1 gene reduced plaque accumulation and improved cognitive performance (Han et al. 2024; Karimian et al. 2020; Mehdizadeh et al. 2025a). Using CRISPR-exosomes as a therapy for PD will alter the SNCA gene mutation that causes α-synuclein accumulation. Thus, it is expected to be available for clinical use within a couple of years (Akyuz et al. 2024a; Kong et al. 2024; Rezaei et al. 2025). In addition, studies have examined how exosomes can be used to deliver CRISPR machinery into cells, enabling targeted treatment of mutations in the LRRK2 gene. Studies have shown that these methods for returning genetic information to cells can successfully restore dopamine levels to normal (Mehdizadeh et al. 2025a; Zhang et al. 2025). Research continues to provide evidence on whether CRISPR and exosome-based delivery methods can target the HTT gene in patients with HD, thereby preventing or slowing disease progression (Akyuz et al. 2024b). Recent studies have shown that exosomes can deliver CRISPR to inhibit the mutant gene responsible for HD, thereby reducing the level of mutant HTT protein. Studies are now being conducted in HD animal models, and the results will determine whether this method is also applicable to human patients (Goel et al. 2025; Purohit et al. 2025).

While CRISPR-exosome therapies show great promise for CNS diseases, several barriers continue to impede their advancement. The primary impediment is concern about heterogeneity in the loading and release of exosome cargo, as well as issues with the efficacy of the various loading/release methods (Horodecka and Düchler 2021). Furthermore, extensive long-term safety studies are required to assess their in vivo implications, even if exosomes exhibit reduced immunogenicity compared to viral vectors (Salomonsson and Clelland 2024).

Research will need to focus on improving the target specificity and functional performance of engineered exosomes through new methods. By incorporating advanced nanotechnology and synthetic biology into the design of engineered exosomes as CRISPR-based tools, these developments could ultimately reduce off-target effects (Bashyal et al. 2022; Ma et al. 2025). In addition, clinical trials can occur only after extensive research, including both preclinical and human studies, has been conducted on the product’s safety and effectiveness (Abeliovich et al. 2020). By developing hybrid CRISPR and exosome-based therapies, researchers are working to create more accurate techniques for treating CNS diseases and to provide a viable vehicle for delivering genetic-editing therapies to the CNS without requiring drugs to cross the BBB. While there are still many technical hurdles to clear before these therapies are ready for prime time, continued progress in both exosome engineering and gene-editing technologies gives researchers hope that these therapies can eventually be used effectively (Duan et al. 2021a; Dubey et al. 2024).

The effectiveness of CRISPR/exosome hybrid systems in treating CNS diseases will depend on their ability to precisely edit genes and cross the BBB. Although exosomes can penetrate the BBB, their size, clearance mechanisms, and targeting strategies limit their effectiveness in delivering CRISPR payloads to the CNS. Recent developments have focused on methods for targeting ligands, combining these exosomes with either NP-based or viral-derived delivery methods to enhance exosome delivery to the CNS (Mehdizadeh et al. 2025b; Sharma et al., 2025). Additionally, studies have examined noninvasive techniques, such as receptor-mediated transcytosis (RMT) and focused ultrasound, to enhance BBB penetration. Thus, to improve CRISPR-exosome hybrid treatment for neurodegenerative and genetic disorders in the brain, it will be critical to develop gene carriers capable of penetrating the BBB. Several nonviral therapeutic approaches are currently available for treating CNS disorders. One common challenge for all non-viral delivery methods is crossing the BBB; therefore, gene carriers must be designed to cross the BBB, enter cells, and be released from cells in a controlled manner. Thus, non-viral delivery focuses on developing gene carriers that adhere to principles for crossing the BBB. In the next section, we detail how engineered gene carriers cross the BBB.

### Emerging Nanocarrier Classes

The main objective of this paper is to provide a comprehensive overview of established non-viral platforms that can deliver drugs via the CNS and contain a significant amount of published literature in this area. Several other nanocarrier classes have been used in drug delivery research (see Fig. 1). However, none of these carriers have yet found their way into the delivery of drugs/gene therapy in the CNS and thus have only recently begun to receive systematic evaluation.

#### Dendritic Systems and Dendrimers

Dendrimers are highly branched, monodisperse polymeric macromolecules that possess tunable surface functionality and internal cavities capable of encapsulating therapeutic agents. Dendrimers have been evaluated as delivery vehicles to improve bioavailability, control drug release, and enhance cellular uptake in peripheral and certain neurogenic tissues, owing to their versatility in size and functional groups. Recent research has described their usage as carriers of neurotheranostics and targeted delivery across the blood-brain barrier in preclinical models, where surface modification has been employed to enhance brain delivery and decrease off-target effects. However, there are limited clinical data supporting the use of dendrimers and dendritic systems for CNS gene delivery and there are few published comparative studies between other non-viral gene delivery vectors (Li et al. 2022).

#### Hydrogel and Nanogel Network

The Hydrogel is a branched three-dimensional polymer network that contains a large amount of water. Hydrogel systems, including hydrogel microspheres and nanogels, have been studied for delivering drugs to the brain. Hydrogel systems have the ability to deliver drugs for long periods of time and can be implanted locally or implanted on-site for long-term use. However, although they have been shown to have a large distribution through a permeable blood-brain barrier (BBB) for drug delivery, these hydrogel-based systems have not yet been widely used for systemic delivery of DNA (Delgado-Pujol et al. 2025).

#### Microsphere and Hydrogel Microspheres

Drug delivery systems using microspheres contain a polymeric or composite matrix that will encapsulate, or package, a drug or biomolecule and then deliver the drug/molecule to a targeted site in a timely and desired manner. The design of drug-loaded microsphere systems is being developed with an emphasis on targeted and sustained therapy; several emerging platforms combine hydrogel and nanoparticle characteristics to modulate release kinetics. Unfortunately, there has been little research about the use of these microspheres for the delivery of nucleic acids/gene editing complexes for CNS applications; additionally, there continues to be very little known about the efficacy of these microspheres for penetrating the BBB compared to lipid and polymer nanoparticle systems (Lee and Kim 2025).

#### Liquid Crystalline Nanoparticles

Cubosomes and hexosomes are examples of liquid crystalline carriers that have a unique internal structure or profile. These structures can provide a high surface area and accommodate a variety of therapeutic agents. Recent research studies indicate that these materials have great potential not only for controlled release of drugs but also as bioadhesive materials that can facilitate targeting to the brain via receptor-mediated transport pathways, though investigations regarding the use of these materials for gene delivery to the CNS is still at a preliminary state (Leu et al. 2023).

#### Niosomes

Niosomes are created when non-ionic surfactants self-organize into vesicles with or without cholesterol. Niosomes are structural analogues of liposomes and have been found to be stable, less expensive than traditional drug delivery systems, and would increase the solubility and bioavailability of drugs. Although niosomes have been studied as a delivery system to the central nervous system through direct nose-to-brain administration approaches, there is still limited evidence of their efficacy as a vehicle for gene therapy across the blood-brain barrier (Gharbavi et al. 2018).

Overall, these platforms provide much-needed design contributions to the field of nanocarrier design relative to many existing designs; however, they do not yet offer the same depth or breadth of mechanistic, translational, and comparative data. Therefore, adding these structures to Fig. 1 serves only as examples of future directions and architectural variety, with a clear indication of the absence of extensive data for CNS gene delivery.

## Engineering Blood-Brain Barrier-Penetrating Gene Carriers

The BBB is a significant obstacle to CNS-targeted gene delivery, limiting the efficacy of gene therapy for hereditary and neurodegenerative brain disorders. Several approaches have been developed that leverage receptor-mediated transport, NP optimization, peptide engineering, and focused ultrasound (FUS) to produce gene carriers that efficiently cross the BBB. These advances offer significant opportunities to improve the brain’s uptake of gene therapies. The blood-brain barrier is the major barrier to achieving successful CNS gene therapy. Successful non-viral delivery requires the planned design and/or use of methods that exploit the body’s natural transport mechanisms or that increase permeability for only a brief period (Fig. 3). Ligands (i.e., ligands that bind to), such as transferrin, apolipoprotein E (ApoE), or brain-targeting peptides, have been attached to gene carriers to enhance the receptor-mediated transport (transit) of these carriers through the blood-brain barrier (Haqqani et al. 2024; K. Wang et al. 2025a, b, c). Drugs cross the BBB via receptor-mediated transport (RMT). When a specific ligand binds to the appropriate receptor on the BBB endothelium, it forms a receptor-ligand complex that triggers endocytosis and movement into brain tissues (Haqqani et al. 2024; Thomsen et al. 2022). Interactions with the transferrin receptor, which is highly expressed in endothelial cells of the BBB, have been shown to improve BBB permeability using transferrin-conjugated NPs (Kalluri and LeBleu 2020b). Leveraging ApoE in lipoprotein transport across the BBB, ApoE-modified LNP s have been used to improve gene delivery efficiency (Dal Magro et al. 2017).

NP size optimization is a key aspect of NP BBB penetration and thus influences the efficiency and success of gene-carrier transport to the CNS. Studies show that the permeability of particles below 100 nanometers is higher than that of larger particles (Saraiva et al. 2016a; Xu et al. 2023). Small NPs can help the transmigration of cellular components by binding to receptor sites on endothelial cells. They can also pass directly through tight junctions formed by two adjacent endothelial cells. It has been shown that polymeric NPs with a diameter of 50 nm exhibit increased transcytosis, thereby facilitating their passage across the blood-brain barrier and into CNS tissue(Ribovski et al. 2021; Saraiva et al. 2016a). Non-viral vector sizes correlate with a diverse tissue penetration pathway (affording non-viral vectors the ability to travel through tissues from the blood vessel capillary pathways to the individual cells). Because they are larger than adenoviral vectors, on average, non-viral vectors have longer blood circulation times, allowing greater CNS absorption than adenoviral vectors (Lu et al. 2023; Xie et al. 2023).

A practical method peptide-mediated transcytosis. Recently, advances in their use in gene transfer into the brain (Prades et al. 2025; Sánchez-Navarro y Giralt 2022). Peptides from BBB endothelial cells can attach these molecules (i.e., NSPs, or NPs) to add targeting/increased internalization effects when linked via NPs to BBB endothelial cells and/or to receptors on the BBB (i.e., through modulation of receptor binding to ligands). The use of cell-penetrating peptides (e.g., TAT (trans-activation of transcription) or RVG (rabies virus glycoprotein) in conjunction with methods such as transcytosis has been reported to deliver nucleic acids across the BBB into the CNS. Recently, new classes or families of peptide sequences derived from natural neurotropic viruses have been designed with enhanced ability to cross the BBB without provoking any immune response (Cavaco et al. 2024; Ghorai et al. 2023; Li et al. 2024).

The development of a new type of temporary BBB disruption is enabled by non-invasive FUS, which may facilitate gene therapy delivery by allowing carriers to transiently breach the BBB. When FUS is applied to the brain in conjunction with microbubble-based contrast agents, it creates temporary openings in the tight junctions between endothelial cells of the BBB, thereby permitting the influx of larger gene therapies into the CNS(Kong and Chang 2023; Zhu et al. 2024). In nonviral gene therapy, this method has been highly successful, as it reduces systemic exposure by targeting delivery. Preclinical studies have shown that FUS-induced BBB opening enhances the delivery of plasmid DNA, RNA therapies, and NPs into the brain (Aly et al. 2023; Kwak et al. 2024). Significantly, FUS has been used in conjunction with receptor-mediated targeting to enhance specificity and minimize off-target effects (Timbie et al. 2017).

Despite advances in the use of nonviral vector systems to bypass the BBB, efficient, safe, and sustainable gene transfer into the CNS remains a significant challenge. Compared with viral vectors, nanoparticle- or exosome-based delivery methods are safer, more versatile, and less likely to elicit an immune response. However, they also come at the cost of lower transduction efficiency and transient expression of the gene product. The limitations and advantages of the various methods will influence the continuing development of CNS-targeted gene therapy Research, resulting in a trade-off between patient safety and clinical effectiveness. To accurately evaluate the relative strengths, weaknesses, and limitations of viral delivery versus non-viral delivery, it is necessary to make side-by-side comparisons between various non-viral methods, including nanoparticle-based systems, exosomal systems, and AAV-based systems, with respect to BBB penetration, expression duration (i.e., how long the gene product will be produced), and the potential for eliciting an immune response.

## Comparative Analysis with AAV-Based Approaches

AAV-derived vectors are considered the standard for gene therapy in the CNS because they can transduce both proliferative and quiescent cell types, including neurons. The ability of AAVs to enable robust, stable expression in the CNS provides new therapeutic options for patients with inherited and neurodegenerative CNS disorders. Additionally, AAV serotypes exhibit distinct tropisms for different cell types in the CNS; therefore, researchers can select the appropriate serotype based on the therapeutic target (Drouyer et al. 2024; Ojala et al. 2015; Xu et al. 2024).

Although AAV-based approaches to gene therapy offer many advantages, they also face challenges to wider clinical use and have significant limitations. A considerable barrier to AAV-based gene therapy is the ability of AAV to elicit an immune response against capsid proteins in a subset of treated individuals, which limits sustained, ongoing transgene expression. As such, patients receiving these therapies may require immunosuppressive medications to reduce the severity of the immune response to the AAV capsid (Arjomandnejad et al. 2023; Prasad et al. 2022).

Due to their limited capacity for genetic cargo (approximately 4.7 kb), AAV vectors cannot accommodate the therapeutic genes required for large-scale therapies targeting both neurodevelopmental and degenerative diseases, such as HD and DMD (Kolesnik et al. 2024; Marrone et al. 2022; Mendell et al. 2012). The heterogeneous distribution of AAV across the brain indicates that improved capsids and delivery methods must be developed to enable broad-based, effective transduction (Carneiro and Schaffer 2024; Chan et al. 2017; Ye et al. 2024).

Compared with AAVs, non-viral gene-delivery methods (e.g., exosomes, liposomes, and NPs) offer additional advantages. Generally, these non-viral alternatives are considered safer because they do not pose the same risks as pre-existing immunity and long-term toxicity (Jayant et al. 2016; Panday et al. 2024; F. Wang et al. 2025a, b, c). In addition, in comparison to AAVs, non-viral vectors provide AAVs with significant advantages because they allow AAVs to deliver larger sizes of genetic content (payloads) that may include all of the components of CRISPR/Cas9 and other therapeutic genes. Finally, there has been increased interest in exosomes due to their ability to traverse the BBB unaltered and their capacity to deliver cargo to target tissues when modified by surface ligands (El Andaloussi et al. 2013; Salman et al. 2022; Serrano et al. 2025).

Non-viral delivery technologies face significant challenges, despite their advantages. A vital obstacle remains the effective loading of cargo, as achieving high-yield, consistent integration of genetic material presents substantial technical difficulties. Additionally, non-viral vector technology remains at an early stage of development for commercial-scale production under regulatory frameworks, and further refinement is needed to ensure compliance with applicable regulations for clinical manufacturing. Additionally, the challenges associated with entering the CNS can be addressed through advanced engineering methods (e.g., peptide functionalization or ultrasonic-assisted methods) specifically designed for this application (Bellettato and Scarpa 2018; Ponnusamy and Philip 2025; F. Wang et al. 2025a, b, c).

Findings from many recent studies indicate that the development of non-viral gene delivery technologies will help address current limitations and obstacles in the treatment of CNS diseases using viral vector-based therapies. It is expected that hybrid technologies that combine the strengths of AAV with recent advances in non-viral gene delivery will provide a more effective, safer, and individualized approach to treating various neurological disorders (Taghdiri and Mussolino 2024; Wang et al. 2023).

## Mechanistic Studies of Non-viral Drug Delivery to the CNS

Comparing AAV-based gene delivery with other viral vector approaches has highlighted both the advantages and limitations of viral vector-based therapies for CNS disorders. AAVs allow for efficient transduction and long-term expression of transgenes; however, the issues of immunogenicity, payload size limits, and therefore, these barriers limit their wider use and has generated a lot of interest into the use of non-viral delivery systems because they offer much more versatility in what can be delivered as well as how much and how many times a particular drug can be delivered. Therefore, understanding how non-viral drug delivery works is essential for developing new approaches to address the limitations of viral vector-based therapies (F. Wang et al. 2024, 2025a, b, c).

### Mechanistic Barriers and BBB Transport

The BBB, an endothelial barrier that selectively permits passage into the brain, is a primary obstacle to the delivery of non-viral treatments to the CNS. To achieve successful delivery to the CNS via non-viral means (liposomes, polymer-based NPs, and lipid-based NPs), one must utilize the natural mechanisms of movement (transport) that exist (via the receptors) at the BBB. Targeting ligands affixed to the surface of delivery vehicles significantly increases the uptake and movement (transcytosis) of those vehicles across the BBB. It emphasizes the factors of size, charge, and surface-adsorbed targeting ligand density in determining the delivery system’s overall effectiveness(Yu et al. 2025; Zha et al. 2024).

### Cellular Uptake and Intracellular Trafficking

In addition to crossing the BBB, non-viral delivery systems must also overcome multiple intracellular barriers, as these cells are located deep within neurons and glia. Mechanistic studies suggest that the material composition of the carrier and its responsiveness to various intracellular signals (e.g., changes in pH or redox potential) are the main determinants of these carriers’ ability to escape from endosomes and release the therapeutic payload into the cytosol. This is of particular importance for nucleic acid-based therapies, as these therapeutic products must be able to enter the cytoplasm (in instances involving RNA products) or the nucleus (in cases of DNA constructs) to be effective (Durymanov and Reineke 2018; Yang et al. 2023).

### Exosomes as Mechanistically Distinct Non-viral Vectors

Exosomes are an exciting way to deliver items non-virally. Their source is within the body, and their mechanism is inherently different from that of viral vectors or synthetic nanoparticles. The ability of exosomes to cross the BBB is due to their use of natural transport pathways throughout the body, and they do so without being detected by the immune system. Mechanistic studies show that exosomal membranes contain native proteins and lipids that facilitate both cell recognition by the exosomal membrane and the uptake of nucleic acids (NA) and proteins by the recipient CNS cell. Surface engineering strategies – such as the linking of specific receptor ligands to the exosome surface via genetic modification of the personal exosome donor cells - exist that aid in the application of exosomal delivery systems to specific tissues and targeted locations within the brain (Abdelsalam et al. 2023a; Choi et al. 2022).

### Alternative Routes and Engineering-Based Enhancements

Carrier design research is occurring in parallel with research into alternative CNS entry routes that may allow for complete avoidance of the BBB. For example, intranasal administration can permit access to the CNS via the olfactory and trigeminal nerve pathways, providing a more direct route with less systemic drug exposure than other routes. Several factors influence the efficacy of intranasal drug delivery; particle size, mucoadhesiveness, and stability in the nasal cavity are formulation characteristics that are important in determining its effectiveness (Lochhead and Thorne 2012). Other innovative technological advances, such as peptide functionalization, stimuli-responsive nanomaterials, and ultrasound-mediated disruption of the BBB, represent a range of engineering approaches currently being explored to enhance CNS penetration and enable spatially controlled drug delivery (Saraiva et al. 2016a; Yu et al. 2025).

### Implications for Hybrid and Next-Generation Therapies

In recent years, amid technological advances, we have found that combining viral delivery methods enables the administration of large, safe doses with non-viral vectors, making this a promising avenue for developing therapies for rare neurological diseases. This has led to significant advances in hybrid delivery systems patient (Paul et al. 2022; K. Wang et al. 2025a, b, c).

## **Artificial Intelligence and Optimizing Gene Delivery Platforms to CNS**

There is a challenge with traditional approaches to the design and delivery of gene therapy for patients with CNS disorders. Conventional methods are limited in their ability to cross the BBB, target specific cell types, and elicit minimal immune responses. An extended timeline and extensive resources are required to optimize traditional approaches to gene therapy through trial-and-error. With the emergence of artificial intelligence (AI) and machine learning (ML), however, more efficient and effective methods have become available for discovering, reengineering, or individualizing the design of a gene-delivery vehicle. Furthermore, AI/ML enables efficient extraction and analysis of vast volumes of gene-delivery data, allowing researchers to predict the effectiveness of delivery methods using their own research data(Gao 2024; Hutchinson et al. 2025; Tan et al. 2025).

Advances in AI techniques are reshaping gene delivery by algorithmic modifications to AAV capsid backbones. Algorithms used to determine the tissue-specific tropism of AAV vectors, immune evasion strategies employed against each vector, and protein-protein interactions between the AAV capsid protein and other proteins of interest standardize AAV capsid production targeting the CNS. Using deep learning (DL) to identify AAV capsid variants that are most likely to cross the BBB will enable the development of AAV capsids with a significantly reduced incidence of off-target effects(Fu et al. 2024; Tan et al. 2025). Furthermore, generative AI techniques can produce unique synthetic capsids, extending the range of AAV-based gene therapies beyond naturally occurring serotypes.

AI is also changing how researchers develop non-viral gene delivery methods (with viral vectors a popular option) by enabling the creation of exosomes and NPs. By combining computer models of biological systems with AI capabilities, researchers can better understand the behavior of NPs as they interact with biological obstacles (e.g., the BBB). Using algorithms, researchers can modify essential NP characteristics (size, surface charge, and surface modification) to enhance their ability to penetrate the BBB (Kapoor et al. 2024). Researchers can develop hybrid exosomes by leveraging AI to identify surface ligand sequences that enhance cellular uptake and precision targeting of the CNS. Combining these tools will provide researchers with a rapid, accurate method for evaluating and selecting the optimal non-viral delivery system for research applications.

AI plays a significant role in predicting and reducing immunological responses to genetic therapies. Both AAV and non-viral vectors have been limited in their therapeutic potential by unintended immunological reactions. This has led researchers to use AI-driven immunogenicity prediction systems (e.g., those from the U.S. National Institutes of Health’s National Cancer Institute) to analyze sequence data and host immunological interactions, thereby informing modifications to gene carriers that protect these vectors from immune detection while maintaining high transduction efficiency (LeMieux 2019). The above statement applies only to AAVs that have been modified and to those designed to deliver specific genes. In addition, owing to their small size and large surface area, LNP scan encapsulate or deliver large quantities of genetic material, making them more effective than traditional carriers (such as plasmid DNA).

The advancement of AI in genetic therapy will enable improved integration of multimodal datasets. AI will integrate single-cell transcriptomics with proteomic datasets and in vivo imaging data to generate refined gene-expression patterns, enabling a consistent, controllable method of transgenic activation within the CNS (Liu 2024; Luo et al. 2025; Nam et al. 2024). Customizing the delivery method of each Gene Therapy based on that individual’s genetic and epigenetic information will provide for more effective treatment outcomes.

The advancement of AI in optimizing gene delivery systems entails significant disruption; however, we are currently in the early stages of AI’s optimization of these systems. In addition, numerous challenges in the use of artificial intelligence to develop gene therapy systems (e.g., data bias, model interpretability, and computational limitations) must be addressed to enhance the reliability and practical utility of these systems. However, as AI continues to advance, we can expect new opportunities to overcome current challenges in gene delivery to the CNS and to facilitate the rapid development of safer, more effective treatments for AD, HD, and other hereditary illnesses(Danaeifar and Najafi 2025; Hasanzadeh et al. 2022).

## Translational and Regulatory Considerations

### Recent Developments in Clinical Trials

Due to the BBB, the brain’s immunological sensitivities, and the complexity of neural networks, gene therapy for the CNS presents unique challenges compared with other areas of medicine. Although numerous non-viral gene delivery technologies have been developed as safer and more flexible alternatives to current viral vector technologies, including AAVs, there is still a role for viral vectors in future developments of gene therapies for neurological disorders. Currently, several clinical trials are evaluating the utility of non-viral gene delivery platforms, including LNPs, polymeric carriers, exosomes, and peptide-functionalized nanocarriers, for the treatment of various neurological disorders.

The use of LNPs for delivering mRNA vaccines against COVID-19 has prompted extensive research into their application for the treatment of neurodegenerative diseases such as PD, ALS, and AD. In particular, a Phase I and II clinical study is using receptor-mediated transcytosis to evaluate the use of LNPs to deliver mRNA to people with ALS, thereby producing neuroprotective proteins. Because LNPs can be engineered to cross the BBB, they will significantly enhance gene delivery to glial cells and neurons(Moosavi et al. 2025; Tashima 2020; Wang et al. 2025a, b, c).

ALN-HTT02 is a new investigational therapy (NCT06585449) designed specifically to reduce the production of the “huntingtin” protein found in the bodies of people suffering from Huntington’s disease by acting on the genetic material that produces it. The safety and effectiveness of this treatment will be assessed using Phase 1 human subjects. (Farag et al. 2025).

With their low immunogenicity and ability to cross the BBB, exosomes are strong candidates for the development of neurological therapies. Product development efforts are ongoing, with completion of a Phase 1b/2a study of an exosome treatment derived from neural stem cells, which was approved by the FDA and is scheduled for listing on ClinicalTrials.gov (NCT number pending assignment). In the preclinical setting, additional research has shown that exosome microparticles containing specific miRNAs enhance the neurorestorative effects of those miRNAs in stroke models, suggesting the great potential of naturally occurring non-viral techniques for introducing miRNAs into the CNS to elicit neuroprotective effects. Recent advances in exosome technology have enabled the development of a potential therapeutic platform for non-viral gene therapy, leveraging exosomes as delivery vehicles, similar to approaches used in mRNA vaccine development(Kudpaje et al. 2025; Yang et al. 2025). Exosomes have the potential to deliver genetic material (miRNA, mRNA, siRNA) to the CNS via the BBB using a noninvasive, easy-to-administer method. Because exosomes naturally cross the BBB, they are being explored for their potential to deliver genes across the BBB and are being investigated as a safe and effective alternative to viral gene-delivery methods.

To date, several studies have shown that miRNA delivered via exosomes exhibits neuroprotective effects in preclinical stroke models; however, research in this area remains in its early stages, and clinical studies are limited. There is currently an FDA-approved exosome therapy (under investigation) available to patients who have had an acute ischemic stroke and who have entered into early-phase clinical trials; however, the formal trial registration process is still ongoing. These recent developments suggest that exosomes may offer a highly selective and low-risk approach to gene therapy within the CNS (Abdelsalam et al. 2023b; Sun et al. 2025).

Over the past couple of decades, peptide-engineered nanocarriers have led to the development of brain-penetrating peptides that may enhance permeability across the (BBB for gene therapy targeting both PD and AD (Poudel and Park 2022).

RVG (rabies virus glycoprotein)-tagged nanoparticles are being studied in clinical trials. Nanoparticles tagged with RVG provide high transfection efficiency and selective delivery of genetic material to neuron-like cells without affecting other cell types. Moreover, different neuron types have varying optimal amounts of RVG-coated nanoparticles for transfection; therefore, determining the most effective dose to achieve maximal transfection efficiency with minimal or no collateral toxicity will yield the best possible transfection outcomes(Chung et al. 2020; Wang et al. 2021).

Despite the promise of non-viral gene delivery, numerous challenges remain, including efficient cargo loading, large-scale production, and sustained transgenic expression in CNS tissues. Clinical translation remains a young discipline; continued development and research are essential to achieve greater stability of nanocarriers, enhance the Precision of delivery for therapeutic agents, and improve the efficacy of genome-based therapy vectors. Future research and development in clinical translation are likely to employ computer modeling and AI to improve the design of gene carriers and enhance their clinical utility (Kapoor et al. 2024).

Gao et al. (2024) compare and contrast therapeutic modalities (rigorously separated) and them within the CNS treatment area, employing an analysis based on research data compiled from historical findings and other sources. More importantly, they outline the known potential benefits of gene therapy as a therapeutic alternative for neurological disorders, while also identifying several limitations associated with this approach. Present current technologies for administering gene therapies, including a brief discussion on novel methods for administering gene therapies to treat diseases of the CNS. This review examines how to address the problem of delivering genetic material (gene therapy) across the BBB. The authors emphasize that no systems currently exist to deliver therapeutic genes to specific regions of the CNS effectively, underscoring the need to develop new gene-delivery methods. This document presents existing methods and emerging techniques the targeted delivery of therapeutic genes to various CNS cell types, including neurons and glia. The study focuses on identifying methods that minimize the risk of off target effects and examines the challenges of achieving efficient gene transfer into neurons and glial cells. In particular, the authors point out several weaknesses in the current state of gene therapy that may limit its use for CNS disorders, including vector immunogenicity, manufacturing scale-up, and interspecies variation in responsiveness to gene therapy. The translational barrier must be overcome gene therapy to be effectively used for CNS disorders. The past clinical trials reviewed in this study have provided the authors with vital information to guide future research on the development of gene therapies for CNS diseases. The publication’s message is clear: improving gene delivery technologies and vector design will ultimately create a coherent strategy that addresses the current limitations of gene therapy methods, delivery systems, and the methods used to deliver them(Gao et al. 2024; Liu et al. 2025).

In an effort to provide a balanced perspective on patients with PD, Currie et al. (2024) examined novel technologies to address mobility and other PD-related symptoms and to develop therapeutic options that had not previously existed. An overview of neuromodulation techniques was presented, highlighting a technique called temporal interference (temporal interference is an advanced form of non-invasive neuromodulation that interrupts or alters brain activity via electrical stimulation). The temporal interference technique enables direct access to the neuromodulation site while leaving nearby areas unaffected. In addition, nanoparticles were evaluated throughout the entire article to facilitate the delivery of PD medications. The morphological and functional characteristics of magnetothermal, up conversion, magnetoelectric, and ultrasound-responsive (magnetothermal/ultrasound) drug delivery nanoparticles were described in detail. Because the BBB restricts the penetration of systemic drugs into the brain, nanoparticles will be required for PD treatment. Another area of exploration within the article is the temporary opening of the BBB using high-intensity ultrasound. By temporarily creating an opening in the BBB, it is possible to allow drugs to gain entry into the brain and, thereby, improve their effectiveness. Gene therapy for the treatment of PD using GABA and dopamine was discussed. The exploration of developing dendritic structures with optimal activation kinetics, or those thermogenically directed, represented an innovative approach to restoring function in PD neurons. Use of DREADDs (Designer Receptors Exclusively Activated by Designer Drugs) to treat PD was also discussed in the article. DREADDs use designer compounds to activate neuron-specific receptors, enabling selective targeting and modulation of PD-related neurons and providing novel control over PD symptoms. Following careful consideration of clinical and preclinical data from many studies, the authors believe they have developed sufficient evidence to design PD therapies based on neuromodulation principles. Finally, the authors point out that their approach to neuromodulation for PD will revolutionize treatment and expand patient access to these therapies(Currie et al. 2024).

The research of Akçimen et al. (2023) indicates advances in understanding the genetics of ALS. Due to increased collaboration among scientists and advances in sequencing technology, researchers have identified numerous genetic variants associated with ALS. Thus, by identifying new genetic variants associated with ALS, scientists will be able to elucidate the pathways that lead to the development of molecularly targeted therapies for ALS. Moreover, scientists have become aware that genetic variation is not uniform across individuals and ethnic groups; therefore, it is essential to understand how specific genes interact with environmental factors to develop new therapeutic options for patients with ALS. The development of a synergistic model that integrates all genetic variants worldwide, along with environmental influences, will provide clinicians and researchers with a comprehensive framework for understanding the causes of ALS and for exploring potential avenues for precision medicine. This also enables translating current knowledge of genes associated with ALS into therapies that can be delivered to patients with ALS(Akçimen et al. 2023).

New technologies that advance nanoparticle design, targeting strategies, and regulatory processes have created numerous opportunities to develop safer, more effective gene therapies. Results from multiple clinical trials indicate that non-viral gene therapies for CNS diseases are advancing rapidly and are effective and safe. Gene therapies for neurodegenerative and genetically induced CNS diseases will be guided by the continued evolution of nanotechnology, molecular engineering, and precision medicine, thereby enabling effective, tailored therapies for these disorders (Currie et al. 2024; Elsabahy et al. 2011; Mohammapdour and Ghandehari 2022; Tenchov et al. 2021).

### Regulatory Challenges

The regulatory environment for non-viral gene delivery technologies (such as AAV) is complex, and both legal and technical barriers hinder practical application within the CNS. The immunogenicity of the vector must be reduced, and the limitations of viral vector systems must be overcome(Alavi et al. 2024). Non-viral delivery systems, such as nanoparticles, polymers, and exosomes, offer numerous advantages over traditional viral vectors. This has led to increased interest in using non-viral delivery systems to treat more complex diseases, including genetic, cancer, and neurological diseases; for instance, the development of LNPs for mRNA COVID-19 vaccines demonstrates how non-viral delivery systems can serve as a gene-therapy option (Maruggi et al. 2022). Notwithstanding these advances, regulatory challenges must be addressed to ensure therapeutic efficacy and safety through non-viral gene delivery approaches for CNS diseases.

A significant regulatory hurdle in establishing uniform criteria for non-viral gene therapy systems employing polymers or lipid particles is the stringent requirements for manufacturing and quality control. Careful control of both the content and the behavior (performance) of these non-viral systems is necessary to protect patients in therapeutic settings. In addition, the size, charge, and composition of either polymeric or LNP materials can vary significantly, thereby creating variable distributions of the medicine and/or unwanted immunologic responses in patients(Currie et al. 2024). Regulatory Agencies, including the EMA and the FDA, continue to face numerous challenges in establishing consistency in non-viral vector standards; however, both maintain rigorous standards for the manufacturing processes, evaluation, and approval of gene therapy products. The development of gene therapy products for CNS disorders presents significant challenges for manufacturers, as each product must undergo its own regulatory approval process.

There are many regulatory hurdles to overcome before all non-viral technologies are commercially viable. With respect to CNS diseases, the primary barrier is the BBB, which prevents many therapeutics from entering the CNS(Tasset et al. 2022). To overcome the barriers posed by nonviral gene transfer, innovative approaches to delivering genetic material must be developed. In addition, to enhance permeability across the blood-brain barrier and to promote targeting to the appropriate cell type, many researchers have developed “non-viral” vectors that contain targeting ligands or cytoplasmic delivery peptides, enabling targeted delivery and transfection (Jain 2007). While preclinical evidence suggests that nanoparticles can be transported across the BBB, their clinical use remains pending further bodies due to concerns about prolonged product stability, potential adverse health effects (toxicity), and limited target-area localization (non-specific).

In addition, non-viral gene delivery methods face numerous challenges in regulating gene stability and expression. For example, in CNS disease treatment, the effectiveness of gene therapy depends on maintaining stable gene expression and avoiding immune responses that could compromise the treatment. Therefore, regulatory agencies require the submission of comprehensive preclinical and clinical data demonstrating that non-viral gene delivery methods can elicit sustained therapeutic benefits without adverse immune reactions. Consequently, extensive long-term evaluations and safety testing will be required before a license can be granted for the use of a non-viral gene therapy (Carvalho et al. 2017). Correcting the timing of these assessments also underscores the need for a complete, consistent, & specifically defined analytical framework for these non-viral gene therapy products, since they continue to lack clear guidance comparable to that for viral vector transfer products, as well as the respective information providers to support those definitions.

Aside from technical barriers, several significant legal constraints exist to the following topics: intellectual property, standardization, and clinical trial regulations. While non-viral delivery systems are more challenging to design than their viral counterparts, developments additional complexity to trial designs. As a result of these problems, obtaining regulatory approvals for the marketing of gene therapy products requires substantial clinical trial data and rigorous testing. Because of differences in delivery mechanisms, viral vectors typically follow standard clinical development processes, whereas non-viral methods require alternative formulations, modes of administration, and targeted delivery approaches (Ediriweera et al. 2021). An internationally recognized legal system cannot emerge for these reasons, and, in addition to questions regarding intellectual property rights in the context of nanoparticle-based treatments, this further complicates regulation.

Despite these limitations, non-viral gene-delivery technologies are advancing through innovative techniques, enhanced regulatory standards, and improved nanoparticle designs, thereby developing new approaches to address these barriers(Maruggi et al. 2022). Many factors influence the successful development of gene therapies and their delivery to patients. Most of these factors are ongoing developments in research and development. They will be pursued in collaboration with Academics and Businesses to develop new delivery methods for non-viral gene therapy, leveraging the best combination of Academic and Business Models.

To conclude, while nonviral gene delivery technologies offer advantages such as safety and flexibility compared to viral vectors for gene therapy, legal issues regarding their use for CNS diseases require improvements in the regulation and production of nonviral vectorization technologies to enhance manufacturing uniformity, targeted efficiency, and consistent gene expression. Ongoing research and technological advances now provide numerous opportunities to develop nonviral gene therapy into a viable treatment option for a range of CNS diseases.

## Future Directions

The ongoing development of non-viral gene delivery systems for the treatment of CNS diseases has important implications for enhancing the efficacy, safety, and translational feasibility of these treatments. Research in these areas will inform key aspects of future work on non-viral gene therapy and will continue to address many of the current limitations of this platform.

### Increasing Blood-Brain Barrier (BBB) Permeability

The difficulty of obtaining sufficient genetic material across the blood-brain barrier poses a significant obstacle to the effective use of non-viral gene-delivery methods. As research progresses, there will be an increased emphasis on developing strategies to enhance BBB permeability. Novel delivery systems could include tailored nanoparticles for optimal brain delivery via surface modification, receptor-mediated transcytosis, and/or ultrasound-assisted transport across the blood-brain barrier. By using ligands conjugated to targeted nanoparticles, such as transferrin or angiopep-2, the efficiency of receptor-mediated transport can be improved, thereby enhancing selectivity for drug delivery.

### Improving Nanoparticle Design and Biocompatibility

Enhancing the physical and chemical properties of nanoparticles—such as size, polarity, and other physicochemical characteristics—is key to improving gene delivery. Future versions of these nanoparticles will be developed to exhibit greater stability under physiological conditions and fewer toxic side effects. By creating flexible, patient-specific, biodegradable polymeric/lipid nanocarrier systems, scientists will greatly accelerate clinical translation.

### CRISPR-Cas9 Genome Editing Integration

Although CRISPR-based genomic editing for CNS diseases is emerging, challenges remain, including precise targeting of the genetic locus, avoidance of off-target effects, and effective delivery. However, technologies may enable accurate, temporary modification of a problematic gene via a combination of CRISPR and non-viral delivery methods, including EXOs and LNPs. The success of developing therapeutics through genome editing for neurodegenerative or genetically based CNS diseases will likely depend on the establishment of exosomal CRISPR delivery platforms that enhance cellular uptake and increase the stability of the therapeutic cargo.

### Exosome Engineering for Targeted Gene Therapy

Exosomes are inherently biocompatible and are emerging as a gene-delivery vehicle, with the potential to penetrate the blood-brain barrier. Future research should focus on large-scale synthesis, loading efficiency, and targeted delivery of exosome-derived therapies. Engineering exosomes to enhance stability, increase loading capacity, and enable targeting of specific tissues via surface modifications will create a scalable and effective gene delivery system.

### Artificial Intelligence-Based Non-viral Gene Delivery Optimization

Machine Learning (ML) and AI have the potential to fundamentally change how non-viral Gene Delivery Systems are designed and optimized. By using Computational Models to predict how nanoparticles will behave, the Stability of the Genes packaged within the Particle, and the penetration into the BBB, AI can further expedite the translation of clinical trials. By using AI-driven methodologies, we can avoid the lengthy, trial-and-error process traditionally associated with product formulation and accelerate the identification of new materials suitable for gene delivery.

### Handling Safety Concerns and Immunogenicity

Although non-viral delivery modalities are less immunogenic than viral vectors, the immunological responses to both synthetic nanoparticles and exosomes pose challenges. Future work should focus on developing immune-evasive delivery methods using patient-derived exosomes, biomimetic coatings, or integrations to minimize immune recognition. Additionally, long-term safety studies should be rigorously evaluated in both preclinical and clinical settings, with attention to biodistribution and potential off-target effects.

### Regulatory Considerations and Clinical Translation

To successfully translate non-viral gene delivery into practice, scaling up and establishing regulatory pathways must be resolved. Future work must establish GMP-compliant manufacturing practices, improve the uniformity of finished products across batches, and develop consistent clinical trial procedures. Regulatory agencies will require extensive data on the safety, effectiveness, and long-term safety of each drug delivery system to support its commercial launch and marketing.

### Customized Precision Medicine Approaches

The advent of Targeted Gene Therapy (TGT) promises to revolutionize personalized treatment, thereby opening new avenues for the use of non-viral gene delivery systems. Now, patients can receive custom-made gene-delivery systems specifically designed to match their genetic makeup, rather than standard treatment options. The omics fields (genomic, transcriptomic, proteomic) will enable the design of patient-specific targeted therapies.

### Review of Final Assessment

The future of non-viral gene delivery methods for CNS Disorders will be determined by of advanced materials science, gene-editing approaches, and computer-aided design and modelling. For these exciting new technologies to be approved for routine clinical application, significant efforts must be made to address issues related to (BBB permeability, immunological responses, and clinical scalability. The introduction of non-viral gene delivery technologies offers an opportunity to change the way patients with neurodegenerative and hereditary CNS disorders are treated by utilizing multidisciplinary approaches and innovations, and, as such, would provide a safer and more efficient method of gene therapy than traditional viral vector-based approaches.

## Conclusion

For gene therapy targeting CNS disorders, non-viral delivery methods (e.g., nanoparticles, exosomes, and lipid-based systems) may represent a viable alternative to traditional AAV-based methods. Benefits of using such approaches include lower immunogenicity vs. AAVs; the ability to engineer enhanced BBB penetration; and the potential for customized delivery to address individual patients’ clinical needs. Therefore, the ability of non-viral delivery systems to meet the clinical needs associated with neurodegenerative diseases, inherited disorders of the CNS, and CNS injuries, by leveraging these advantages, makes them promising tools for their management. Nevertheless, additional studies are necessary to improve existing non-viral gene delivery systems; determine how well these systems can be manufactured on an industrial scale; and to properly evaluate the effectiveness of these therapies. Advances in technology should be expanded to create opportunities for non-viral gene delivery, with the potential to significantly impact the future treatment of CNS diseases and to redefine paradigms in the use of gene therapy for various neurological diseases.

## Data Availability

No datasets were generated or analysed during the current study.

## Abbreviations

AAV: Adeno-associated virus
AD: Alzheimer’s disease
AI: Artificial intelligence
ALS: Amyotrophic lateral sclerosis
AONs: Antisense oligonucleotides
ApoE: Apolipoprotein E
APP: Amyloid precursor protein
Aβ: Amyloid-beta
BBB: Blood–brain barrier
Cas9: CRISPR-associated protein 9
CNS: Central nervous system
COVID-19: Coronavirus disease 2019
CRISPR: Clustered regularly interspaced short palindromic repeats
DNA: Deoxyribonucleic acid
DMD: Duchenne muscular dystrophy
DREADDs: Designer receptors exclusively activated by designer drugs
EMA: European Medicines Agency
EXO: Exosome
FDA: Food and Drug Administration
FUS: Focused ultrasound
GABA: Gamma-aminobutyric acid
gRNA: Guide RNA
GMP: Good manufacturing practice
HD: Huntington’s disease
HDR: Homology-directed repair
HTT: Huntingtin
LDP: Low-density lipoprotein
LNP: Lipid nanoparticle
LRRK2: Leucine-rich repeat kinase 2
ML: Machine learning
miRNA: MicroRNA
mRNA: Messenger RNA
MNC: Magnetic nanocarrier
NNI: Narrow nanotechnology
NP: Nanoparticle
PD: Parkinson’s disease
PEG: Polyethylene glycol
PEI: Polyethyleneimine
PK/ADME: Pharmacokinetics/absorption, distribution, metabolism, and excretion
PLGA: Poly(lactic-co-glycolic acid)
RNA: Ribonucleic acid
RMT: Receptor-mediated transcytosis
RNP: Ribonucleoprotein
RVG: Rabies virus glycoprotein
siRNA: Small interfering RNA
SLN: Solid lipid nanoparticle
SNCA: Alpha-synuclein gene
TAT: Trans-activator of transcription
TGT: Targeted gene therapy
US: Ultrasound
